# Suppression of Disorder in Benzamide and Thiobenzamide Crystals by Fluorine Substitution

**DOI:** 10.1021/acs.cgd.4c00517

**Published:** 2024-06-10

**Authors:** Alexander G. Shtukenberg, Doris E. Braun, Melissa Tan, Noalle Fellah, Bart Kahr

**Affiliations:** † Department of Chemistry and Molecular Design Institute, New York University, New York, New York 10003, United States; ‡ Institute of Pharmacy, Pharmaceutical Technology, University of Innsbruck, 6020 Innsbruck, Austria; § Christian Doppler Laboratory for Advanced Crystal Engineering Strategies in Drug Development, Institute of Pharmacy, University of Innsbruck, 6020 Innsbruck, Austria

## Abstract

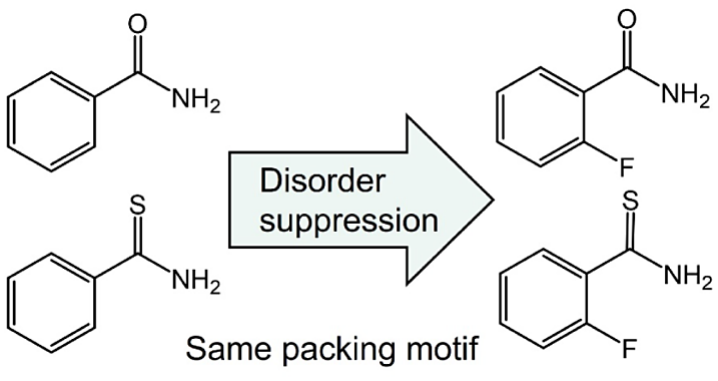

Disorder is a common feature of molecular crystals that complicates determination of structures and can potentially affect electric and mechanical properties. Suppression of disorder is observed in otherwise severely disordered benzamide and thiobenzamide crystals by substituting hydrogen with fluorine in the *ortho*-position of the phenyl ring. Fluorine occupancies of 20–30% are sufficient to suppress disorder without changing the packing motif. Crystal structure prediction calculations reveal a much denser lattice energy landscape for benzamide compared to 2-fluorobenzamide, suggesting that fluorine substitution makes disorder less likely.

## Abstract 2

Disorder is a common feature of molecular crystals that complicates characterization and affects properties. We demonstrate suppression of disorder in otherwise severely disordered benzamide and thiobenzamide crystals without changing the packing motif by substituting hydrogen with fluorine in the *ortho*-position of the phenyl ring. Crystal structure prediction calculations help rationalize this observation.

## Introduction

In a 2004 article called “Organic fluorine: Odd man out,”^1^ Dunitz argued that organic fluorine has been long misunderstood, and that there remains much to be discovered. More recent authors have tended to describe the unanticipated properties of fluorine with fantasy words, although magic is not required.^2^ Even though F atoms have many more electrons than H atoms, and C–F and C–H bonds are oppositely polarized, in suitably dissymmetric compounds, like aryl fluorides with F in the 2 or 3 positions, the aryl ring will often become statically disordered with partial H and F occupancies at a given position, perhaps because H and F have similar polarizabilities. In any case, substituting aryl F atoms in place of aryl H atoms, symmetry permitting, often tends to disorder.^3^

Lately, we have been distracted by crystal structures of benzamide foremost because a metastable form that grows from hot aqueous solutions invariably deposits fibers with twisted habits.^4^ However, by virtue of the tendency of the metastable polymorphs of benzamide to be polytypic, we have been motivated to make a comprehensive evaluation of imperfect benzamide crystal structures.

**Scheme 1:**
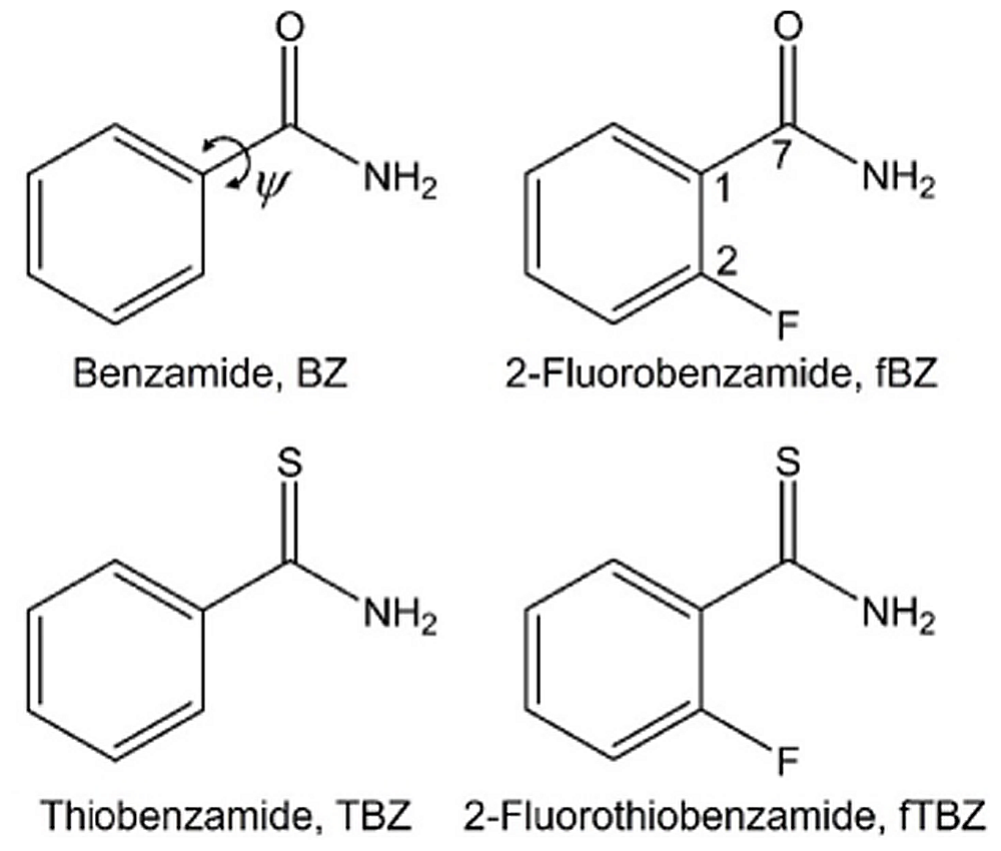
Molecular Structures of Select Benzamide Derivatives The O–C–C–C torsion angle ψ is shown for benzamide.

**Figure 1:**
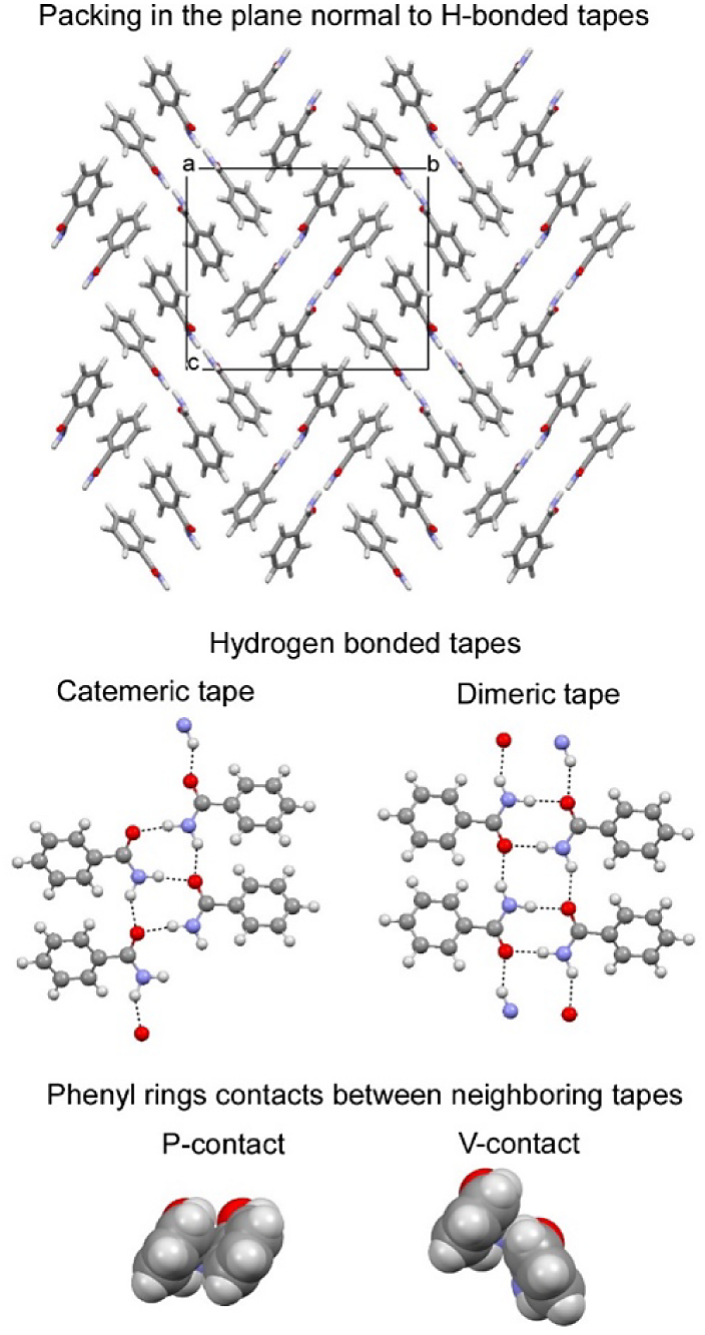
Crystal structures of benzamide derivatives with two parallel hydrogen-bonded tapes. The in-plane packing is shown for the *P*2_1_/c candidate structure for benzamide **II** reported in ref.^13^.

Benzamide (Scheme 1) is the first discrete molecule, for which two different crystalline forms (polymorphs) were discovered.^5,6^ In 1832, Liebig and Wöhler obtained needlelike crystals by slow crystallization of hot benzamide aqueous solutions and observed their transformation to the stable polymorph.^7^ Unlike stable polymorph **I**,^8,9^ it was impossible to obtain crystals of metastable polymorph **II** suitable for single crystal X-ray diffraction analysis. Its crystal structure was instead solved from powder diffraction data in 2005 in the space group *Pba*2.^10,11^ Eleven years later, the structure was reexamined with the same data and crystal structure prediction (CSP) methods. It was concluded that the original solution was not likely due to high lattice energy (recently, we came to the same conclusion^12^). Instead of the *Pba*2 solution, two other low lattice energy structures, *Fdd*2 and *P*2_1_/*c*, were suggested that matched the data.^13^ The main structure fragments of all three models of benzamide **II**, as well as of benzamide **I** and later discovered polytype **III**,^14−16^ are dimeric or catemeric tapes running along a direction with a periodicity of *ca*. 5 Å. Two parallel tapes constitute what we shall refer to as “tiles” in cross-section in all models for benzamide **II**, contributing in-plane to lattice constants that are multiples of 14.7 × *m* and 17.4 Å × *n* (Figure 1), where *m* and *n* are small integers, 1 and 2, respectively. However, a unique structure and symmetry for the tapes in benzamide **II** was not established due to the evident disorder revealed in cryo-TEM selected area electron diffraction images.^4^ Our recent computer simulations support dimeric tapes with edge-to-face phenyl ring contacts (“V-contacts”, Figure 1) as the main structure element of disordered benzamide **II**.^17^

A few years ago, we discovered a new polymorph, benzamide **IV** by crystallization from the melt.^12^ Again, only powder X-ray diffraction data were collected. Due to even stronger disorder, the diffraction pattern contained only *hk*0 reflections and exhibited significant diffuse scattering. A combination of simulated annealing, CSP, and DFT optimizations supported several candidate structures similar to benzamide **II**,^12^ consisting of catemeric tapes with a 5 Å periodicity, but had slightly different lattice constants in the perpendicular direction that corresponded to the multiples of 11.8 and 20.5 Å. The precise structure was again not established. Of the four known crystalline polymorphs of a comparatively simple compound, two of them, **II** and **IV**, resisted the reduction to a single structure, burdened as they were with disorder and/or polytypism.

To obtain initial structural models for benzamides **II** and **IV**, we searched for derivatives composed of pairs of hydrogen-bonded dimeric or catemeric tapes arranged in a parquet-like motif normal to the *ca*. 5 Å period. Six such molecules were identified. Thiophene-2-carboxamide (refcodes TUKPOF and ZAZZEI), thiophene-3-carboxamide (refcode HUZGOB),^18^ and pyridine-2-carboxamide (picolinamide, refcodes PICAMD01, PICAMD03, PICAMD05) are flatter than benzamide, with the O–C–C-C torsion angle ψ (Scheme 1) generally <10°, compared with ψ = 23–38° for benzamide (Table 1). Their solid-state structures showed substantial differences compared with the motifs previously illustrated as candidate structures for benzamides **II** and **IV**.

**Table 1:** Features of Benzamide Derivatives that Adopt Double Tape Motifs in their Crystal Structures

compound, space group	torsion angles, ψ, deg	H-bonded tapes	phenyl ring contacts^c^	ref(s)
**BZ**, form **II**, *Pba*2^a^	24–30	↑↓ dimers	P + P	(10), (11)
**BZ**, form **II**, *Fdd*2^b^	23–27	↑↑ catemers	V + V	(13)
**BZ**, form **II**, *P*2_1_/*c*^b^	25–29	↑↑ dimers	V + V	(13)
**BZ**, form **IV**, *Fdd*2, *P*2_1_2_1_2^b^	26–38	↑↑ catemers	V + V	(12)
**fBZ**, *P*2_1_/*c*	29–35	↑↑ dimers	V + V	(24), our data
**BZ-fBZ**, *Pna*2_1_	28–32	↑↓ dimers	P + V	our data
**TBZ**, *P*2_1_/*c*^a^	16–39	↑↑ dimers	V + V	(25)
**TBZ**, *P*2_1_/*c*	20–36	↑↑ + ↑↓ dimers	P + V	our data
**fTBZ**, *P*2_1_/*c*	23–39	↑↑ dimers	V + V	(26), our data
**TBZ-fTBZ**, *P*2_1_/*c*	23–39	↑↑ dimers	V + V	our data

a Unlikely structure due to high lattice energy.

b Plausible packing that alone is not sufficient to describe this very disordered structure.

c P and V contacts are illustrated in Figure 1. The arrows indicate parallel (↑↑) vs antiparallel (↑↓) tapes.

However, 2-fluorobenzamide had similar torsion angles ψ = 29–35° (Table 1). Even though structure-directing properties of organic fluorine in molecular crystals have been recognized,^19,20^ fluorine’s role in establishing a crystal structure is typically weak, as illustrated by isostructural compounds and solid solutions formed with monofluorinated and sometimes difluorinated analogues.^3,21−23^

Here, we aim to understand crystal structures of a nonfluorinated benzamide by systematically studying the effect of fluorination on the *ortho*-position of benzamide and thiobenzamide (Scheme 1). A combination of single crystal and powder X-ray diffraction, as well as computational methods, reveals that even a small incorporation of 2-fluorobenzamide or 2-fluorothiobenzamide suppresses disorder. All these structures adopt a packing with two parallel dimeric/catemeric tapes shown in Figure 1 and later called a double tape motif.

## Experimental Section

Benzamide, **BZ** (99%), 2-fluorobenzamide, **fBZ** (98%), and thiobenzamide, **TBZ** (98%) were purchased from Sigma-Aldrich; 2-fluorothiobenzamide, **fTBZ** (95%), was purchased from Enamine. All chemicals were used without further purification.

Thin films of **BZ**, **fBZ**, **TBZ**, and **fTBZ** as well as of **BZ-fBZ** and **TBZ-fTBZ** solid solutions were prepared by melting 2–4 mg of powder between a glass slide and a coverslip on a Kofler bench. The samples were crystallized either at temperatures close to the melting point by spontaneous nucleation or via seeding, or at room temperature by rapid quenching of the melt between two metal blocks. To slow down crystallization and polymorph conversion, 10–20% of Canada balsam or gum mastic were occasionally added to the melts. Single crystals of **BZ**, **fBZ**, **TBZ**, and **fTBZ** as well as those of **BZ-fBZ** and **TBZ-fTBZ** solid solutions were grown from water, ethanol, or water–ethanol mixtures by temperature lowering or solvent evaporation.

Two-dimensional X-ray microdiffraction (2D μ-XRD) was performed with a Bruker D8 Discover microdiffractometer with the General Area Detector Diffraction System (GADDS) equipped with a VÅNTEC-2000 2D detector and Cu-Kα source (λ = 1.54178 Å). The X-ray beam was monochromated with a graphite crystal and collimated with a 0.5 mm capillary collimator (MONOCAP). The powder was loaded into 0.8 mm Kapton capillaries.

High-resolution synchrotron powder diffraction data were collected at beamline 11-BM at the Advanced Photon Source (APS), Argonne National Laboratory, using an average wavelength of 0.412827 Å. Discrete detectors covering an angular range from −6 to 16° 2θ were scanned over a 34° 2θ range, with data points collected every 0.001° 2θ and a scan speed of 0.01°/s. A few mg of **BZ** and a **BZ-fBZ** mixture (mole fraction of fluorinated moiety *x*_F_ = 0.3) with *ca*. 20 wt % of gum mastic were placed between a microscope slide and a glass coverslip and melted on a Kofler bench at *ca*. 140 °C. The sample was rapidly cooled to room temperature. The coverslip was detached, and the powder was carefully scraped off with a razor blade. The powder was loaded into the Kapton 1.5 mm capillary and measured at 100 K using an Oxford 700+ Cooler.

High signal-to-noise ratio synchrotron powder diffraction data was collected on the 17-BM beamline of the Advanced Photon Source, Argonne National Laboratory, at a wavelength of 0.4524 Å using a PerkinElmer PE1621 area detector. The sample–detector distance was 500 and 1000 mm, and the measurement covered the angular range 2θ = 0.5–22.8°. A few mg of **BZ-fBZ** mixtures (*x*_F_ = 0.4, 0.5, and 0.7) were placed between a microscope slide and a glass coverslip and melted on a Kofler bench at *ca*. 140 °C. The sample was rapidly cooled to room temperature. The coverslip was detached, and the powder was carefully scraped with a razor blade. The powder was loaded into a Kapton 1.0 mm capillary and measured at 100 K using an Oxford Cryosystems 700+ Cooler.

Single-crystal X-ray diffraction data sets were acquired on a Bruker SMART APEX II diffractometer equipped with a CCD detector. The X-ray beam generated from a sealed tube was monochromated with a graphite crystal and collimated with a MONOCAP collimator (Mo–Kα radiation, λ = 0.71073 Å). The crystal temperature (100 K) was controlled by an Oxford Cryosystems 700+ Cooler. Crystals were mounted on a 0.2 mm MicroMount (MiTeGen) with Type B immersion oil (Cargille Laboratories). Data were collected and processed using the APEX2 software (version 2013.12) for data reduction, data correction, and cell refinement.^27^ The structures were solved by SHELXT^28^ and refined with full-matrix least-squares by SHELXL (Sheldrick 2014).^29^ Non-hydrogen atoms were refined with anisotropic displacement parameters, and hydrogen atoms were placed in idealized positions and refined with riding models. Crystallographic data have been deposited with the Cambridge Crystallographic Data Centre, and their corresponding deposit numbers are listed in Table 2. Copies of these data can be requested from, free of charge, the CCDC website at https://www.ccdc.cam.ac.uk/structures/.

**Table 2:** Crystal Structure Refinement Details for Benzamide Derivatives

	**1**	**2**	**3**	**4**	**5**	**6**	**7**
composition	**fBZ**	**BZ-fBZ**	**BZ-fBZ**	**BZ-fBZ**	**TBZ**	**TBZ-fTBZ**	**fTBZ**
formula	C_7_H_6_FNO	C_7_H_6.86_F_0.14_NO	C_7_H_6.61_F_0.39_NO	C_7_H_6.49_F_0.51_NO	C_7_H_7_NS	C_7_H_6.60_F_0.40_NS	C_7_H_6_FNS
*M*_w_, g/mol	139.13	123.65	128.15	130.31	137.20	144.40	155.19
space group	*P*2_1_/*c*	*P*2_1_/*n*	*Pna*2_1_	*Pna*2_1_	*P*2_1_/*c*	*P*2_1_/*c*	*P*2_1_/*n*
*Z*, *Z*′	8, 2	4, 1	16, 4	16, 4	16, 4	8, 2	8, 2
*a*, Å	5.1256(4)	5.0443(7)	23.850(3)	23.8767(13)	17.859(5)	5.8878(4)	5.8953(3)
*b*, Å	20.3676(16)	5.4336(8)	5.1299(7)	5.1320(3)	5.7850(14)	17.7677(11)	17.5759(9)
*c*, Å	12.3220(10)	22.245(3)	20.486(3)	20.5746(11)	25.817(6)	13.4390(9)	13.8843(7)
β, deg	96.6945(13)	90.664(2)	90	90	90.057(3)	101.6186(9)	100.3616(8)
*V*, Å^3^	1277.60(18)	609.66(15)	2506.5(6)	2521.1(2)	2667.2(11)	1377.08(16)	1415.16(12)
*D*_c_, g/cm^3^	1.447	1.418	1.293	1.371	1.367	1.367	1.457
μ, 1/mm	0.118	0.107	0.097	0.103	0.382	0.377	0.389
2θ range, deg	1.94–28.32	1.83–28.29	1.71–27.14	1.71–28.29	1.39–25.07	1.93–28.30	1.89–28.31
*T*, K	100(2)	100(2)	100(2)	100(2)	100(2)	100(2)	100(2)
total reflns.	3174	1517	5571	6260	4714	3412	3524
obs. reflns. [*I* > 2σ(*I*)]	2386	1407	5172	5544	3977	2932	2968
no. of parameters	181	95	365	366	326	207	217
no. of restraints	0	1	5	1	3	6	5
*R*_1_ [*I* > 2σ(*I*)]	0.0439	0.0523	0.0359	0.0403	0.0827	0.0381	0.0360
*wR*_2_ [all data]	0.1410	0.2100	0.1040	0.1183	0.2030	0.1271	0.1005
GoF	0.938	1.891	0.854	0.878	1.102	1.003	1.044
Flack	n/a	n/a	0.4(3)	0.3(3)	n/a	n/a	n/a
deposition number	2339530	2339536	2339532	2339531	2339534	2339533	2339535

Potential energy surface scans for **fBZ** employed Gaussian09^30^ at the PBE0/6-31G(d,p) level of theory. The dihedral angle N–C7–C1–C2 (Figure 1), was systematically scanned in 15° increments. The global and local minima served as input conformations for *Z*′ = 1 and 2 crystal structure prediction (CSP) studies. Rigid-body CrystalPredictor v2^31−33^ searches covered the 59 most common space groups, generating 8.5 × 10^6^ structures. All structures within 20 kJ mol^–1^ of the global minimum were reoptimized with DMACRYS^34^ and CrystalOptimizer v2.4.8,^35^ using a distributed multipole representation of the charge density^36^ and considering the N–C7–C1–C2 dihedral. Conformational energies and distributed multipoles were calculated at the PBE0/6-31G(d,p) level using Gaussian09. Intermolecular forces used an atom–atom *exp-*6 form with the FIT potential.^34,37^ The lattice energies of the 340 lowest energy structures were recalculated using CASTEP v20.11^38^ (PBE generalized gradient approximation exchange-correlation density functional,^39^ ultrasoft pseudopotentials,^40^ MBD* dispersion correction,^41^*k*-points spacing of 2π·0.07 Å^–1^, basis set cutoff of 780 eV, and convergence criteria: < 2 × 10^–5^ eV per atom, atomic displacements <1 × 10^–3^ Å, maximum forces <5 × 10^–2^ eV Å^–1^, and maximum stresses <0.1 GPa). To ensure comparability between the **BZ** and **fBZ** lattice energy landscapes, the last step was repeated for the lowest energy **BZ** structures.^17^

The experimental and computationally generated structures were initially compared using COMPACK^42^ and the standard settings of Mercury (a cluster of 15 molecules). In the next step, adjustments were made to account for the 180° flip of the amide, meaning that the amide group orientation was ignored.

## Results and Discussion

**Figure 2:**
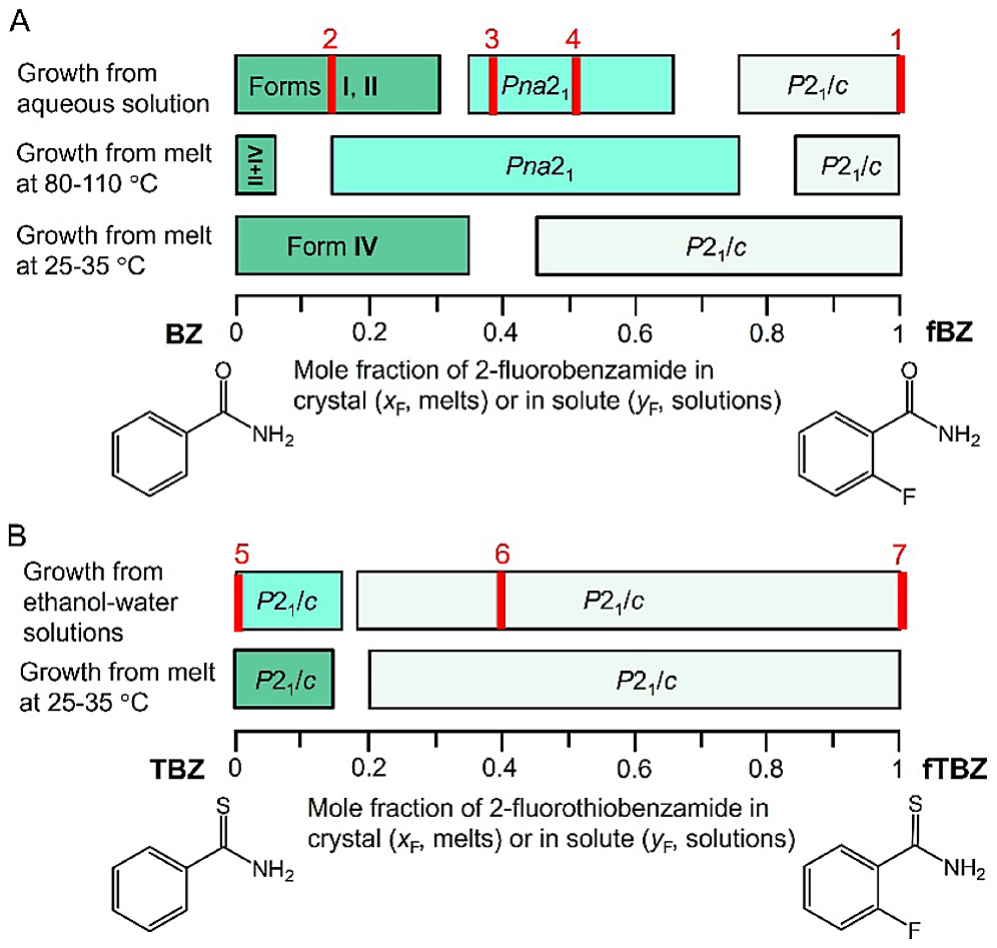
Polymorphism in benzamide – 2-fluorobenzamide (A) and thiobenzamide – 2-fluorothiobenzamide series (B) as revealed by a combination of X-ray diffraction methods. Panel A does not include a second polymorph from the melt observed through the whole range of compositions and corresponds to benzamide **III** at *x*_F_ close to 0. Intensity of color increases as disorder intensifies. Red bars indicate compositions for which single crystal structure determinations (red numbers correspond to structure numbers in Table 2) have been performed.

**Figure 3:**
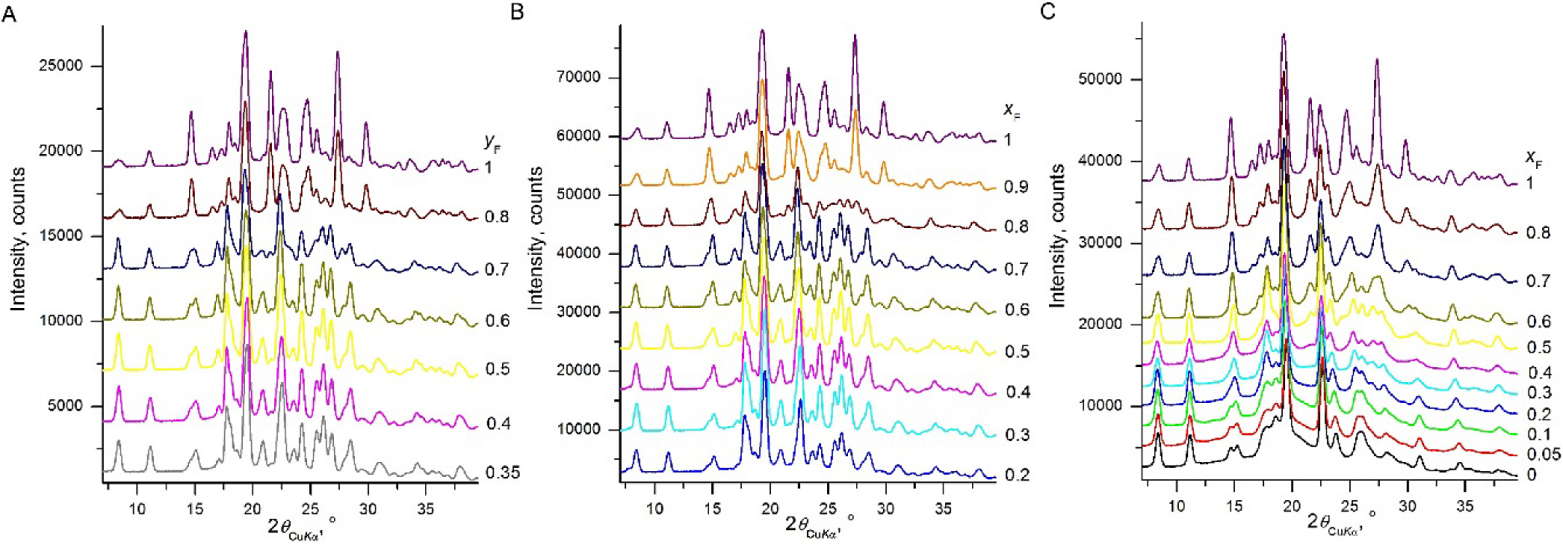
X-ray powder diffraction patterns of benzamide – 2-fluorobenzamide solid solutions. Crystallization from aqueous solutions (A), from melt close to the melting point (B), and from a melt at room temperature (C). *x*_F_ and *y*_F_ are fractions of **fBZ** in the crystal and solute, respectively.

The following mixtures are described by the fluorinated mole fraction in the crystal, *x*_F_, and in the solute, *y*_F_. Benzamide forms a continuous series of solid solutions with 2-fluorobenzamide (Figure 2). The crystal structure of **fBZ** exhibits a double tape packing motif and is composed of parallel dimeric tapes and V-contacts between phenyl rings of the neighboring tapes (Table 1). **BZ**, when crystallized from water or ethanol with small amounts of **fBZ** forms solid solutions with benzamide in form **I** (structure **2** in Table 2). Benzamide **III** was detected only from **BZ** solutions that did not include **fBZ**. At higher supersaturation, needlelike twisted crystals of benzamide **II** can form as well, at least up to the solute molar fraction of **fBZ***y*_F_ ≈ 0.3. For *y*_F_ ≈ 0.3–0.7, a new intermediate compound forms (structures **3** and **4** in Table 2). At *y*_F_ > 0.7, the intermediate is replaced by the crystal structure of **fBZ** (refcode BIGSUF, Table 2, Figures 2 and 3A). In comparison, the structure of **BZ-fBZ** solid solutions (structures **3** and **4** in Table 2) is more complex: antiparallel dimeric tapes and both P and V-contacts between phenyl rings and four different occupancies of a fluorinated moiety for four independent molecules in the unit cell (Table 1).

Melt crystallization close to the melting temperatures produced a similar sequence of phases with the exception that at *x*_F_ < 0.2, benzamide **II**, **III**, or **IV** formed (Table 2, Figures 2 and 3B). Highly disordered benzamide **IV** crystallized near room temperature and at *x*_F_ < 0.4. At *x*_F_ > 0.4, the crystal structure of **fBZ** was observed (refcode BIGSUF, Table 2, Figures 2 and 3C). High-resolution PXRD patterns were collected at APS beamline 11-BM for **BZ** and **BZ-fBZ** solid solution (*x*_F_ = 0.3) crystallized from the melt at room temperature. All *hkl* reflections with *l* ≠ 0 in these patterns were broad and weak, suggesting severe disorder. High signal-to-noise PXRD data were collected at APS beamline 17-BM for **BZ-fBZ** solid solutions (*x*_F_ = 0.4, 0.5, and 0.7) crystallized from the melt at room temperature. These data were fit to the crystal structure of **fBZ**.

**Figure 4:**
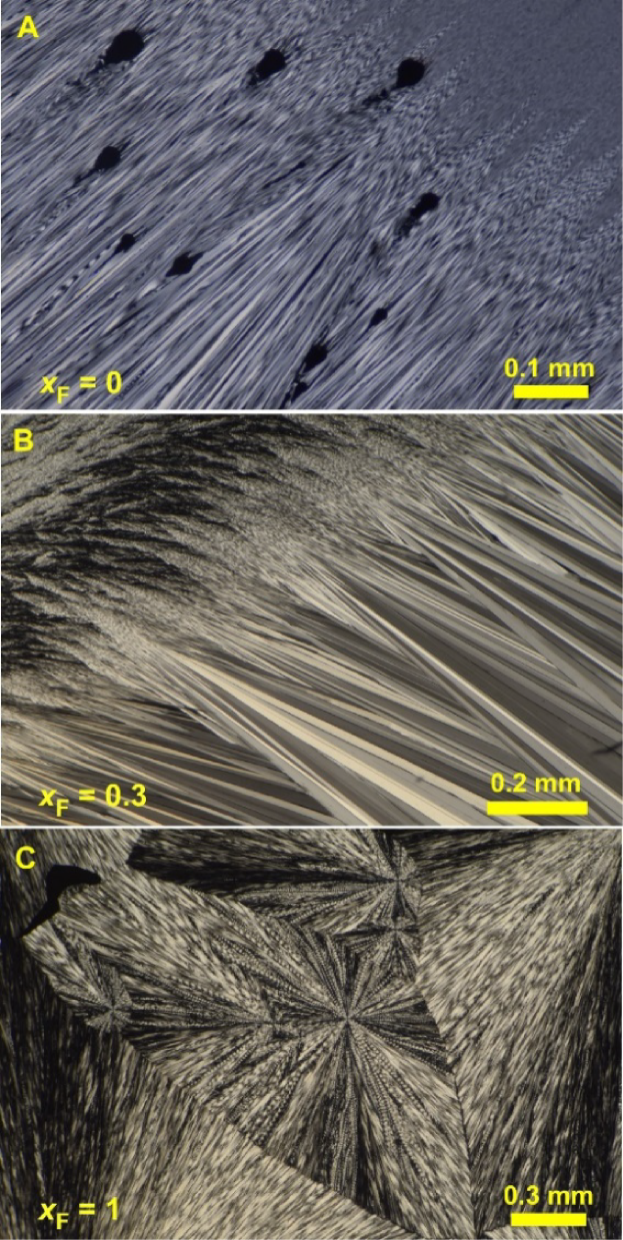
Polarized light optical micrographs of melt crystallized benzamide – 2-fluorobenzamide solid solutions. (A) Spherulites of benzamide **IV** with fine straight fibers crystallized at 25–35 °C and continued growth at 60–90 °C as a mixture of more coarse straight fibers of benzamide **IV** and twisted fibers of benzamide **II**. (B) Fine twisted fibers crystallized 25–35 °C and continued growth at 60–90 °C as coarse straight fibers of the *Pna*2_1_ phase (structures **3** and **4** in Table 2). (C) Spherulites of 2-fluorobenzamide with twisted fibers crystallized at 30–50 °C.

From the melt, the aforementioned compositions (*x*_F_ = 0–1) form optically positive, low-birefringent spherulites, which can also be helicoidally twisted over the whole range of crystal compositions. Twisted morphologies may form in *ca*. one-quarter to one-third of melt-cooled molecular crystals.^18,43,44^ The origins of these nonclassical morphologies – typically incompatible with 3D crystal periodicity – are starting to come into focus.^45^ Twisting was observed only for benzamide **II** (grown from solution^4^ and melt) and crystals with the structure of 2-fluorobenzamide, while structurally similar benzamide **IV** and **BZ-fBZ***Pna*2_1_ phases (structures **3** and **4** in Table 2) grow straight (Figure 4A–C). In the whole range of compositions (*x*_F_ = 0–1) from the melt, one can also obtain coarse low-birefringent spherulites of a second polymorph, which for *x*_F_ = 0 corresponds to benzamide **III**. At higher fractions of 2-fluorobenzamide, this polymorph was not identified because of its small percentage and high metastability.

**Figure 5:**
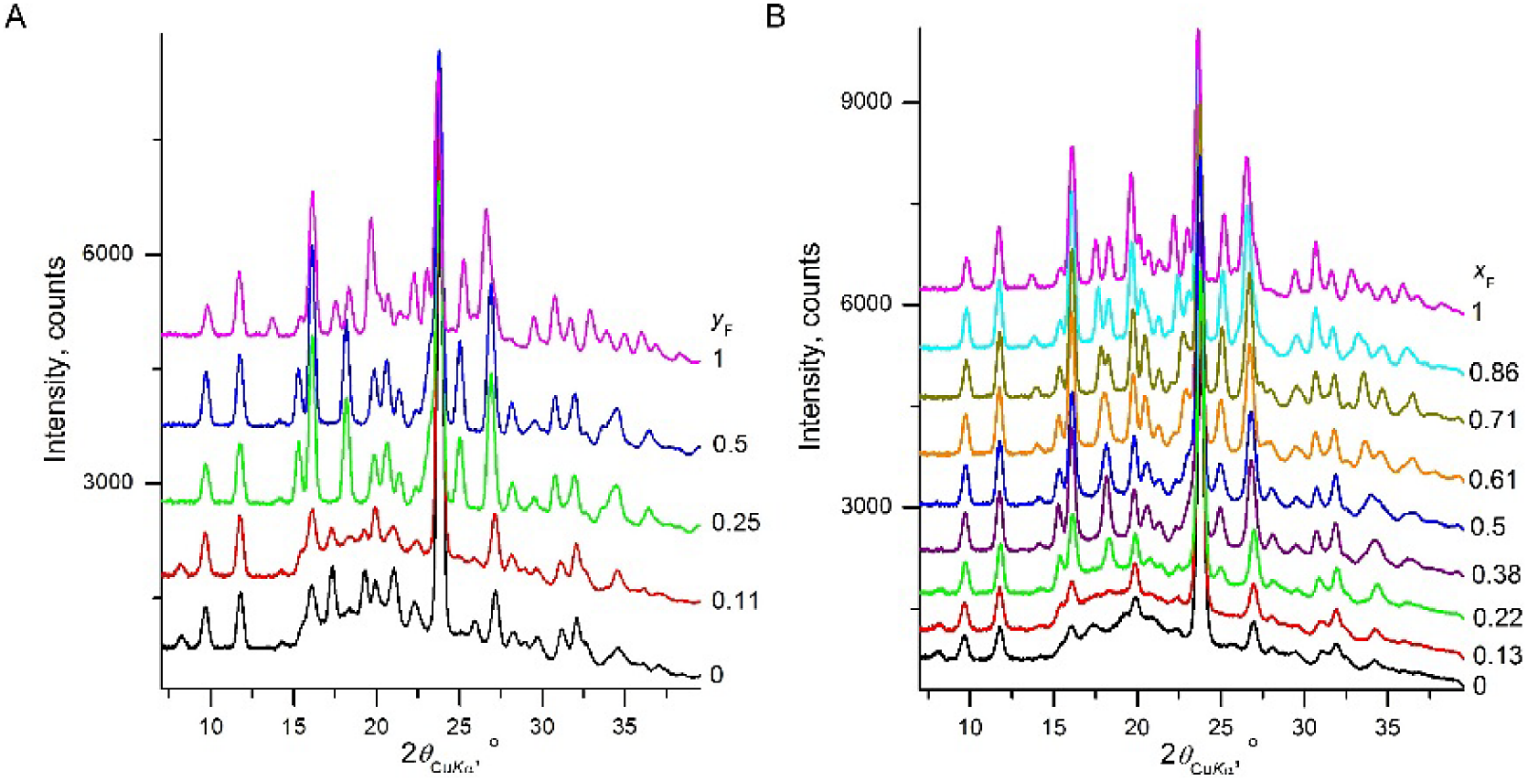
X-ray powder diffraction patterns of thiobenzamide – 2-fluorothiobenzamide solid solutions. Crystallization from water–ethanol solutions (A) and from a melt at room temperature (B). *x*_F_ and *y*_F_ are fractions of **fTBZ** in crystal and solute, respectively.

We turned to thiobenzamide (**TBZ**) to establish what might be the effect of admixing 2-fluoro atoms in solid solutions. **TBZ** is monomorphic, even if crystallized from solution, melt, or vapor phases.^26^ The published crystal structure,^25^ however, did not suitably match our PXRD data, and a new data set was collected from a single crystal grown by sublimation. The redetermined structure was better matched to the PXRD data. The literature structure and our redetermination were comparable but with some differences. Both structures showed the same double tape packing motif. The literature structure exhibited only parallel P-contacts, while our redetermined structure exhibited both parallel and antiparallel P and V-contacts between the phenyl rings (Tables 1, 2). Even though the **TBZ** crystals were not disordered, they formed very thin needles and were severely twinned. This complicated the choice of a structure for diffraction analysis and indicated a propensity of **TBZ** to disorder. **TBZ** cocrystallized from water–ethanol mixtures with 2-fluorothiobenzamide (**fTBZ**) in all proportions. When the fraction of **fTBZ** in solute was low, the PXRD patterns corresponded to the structure of **TBZ**. In the range of 0.11 < *y*_F_ < 0.25, the crystal structure switched to that of **fTBZ** (refcode XOGRIW, Table 2, Figure 5A), which has the same packing as the **fBZ** structure (Table 1).

A similar situation was observed for crystallization from the melt (Figures 2, 5). For *x*_F_ ≥ 0.22, the PXRD pattern fit well to the **fTBZ** structure. For *x*_F_ ≤ 0.13, the pattern more closely corresponded to the **TBZ** structure, yet was highly disordered, showing an amorphous halo at 2θ > 15° and a severe decrease in intensity of some diffraction peaks (compare Figure 5A,B). This disorder made the agreement with structure **5** in Table 2 less reliable. In the whole composition range (*x*_F_ = 0–1), crystals from the melt form optically positive low-birefringent spherulites with nontwisted fibers, which become finer toward pure **TBZ**.

**Figure 6:**
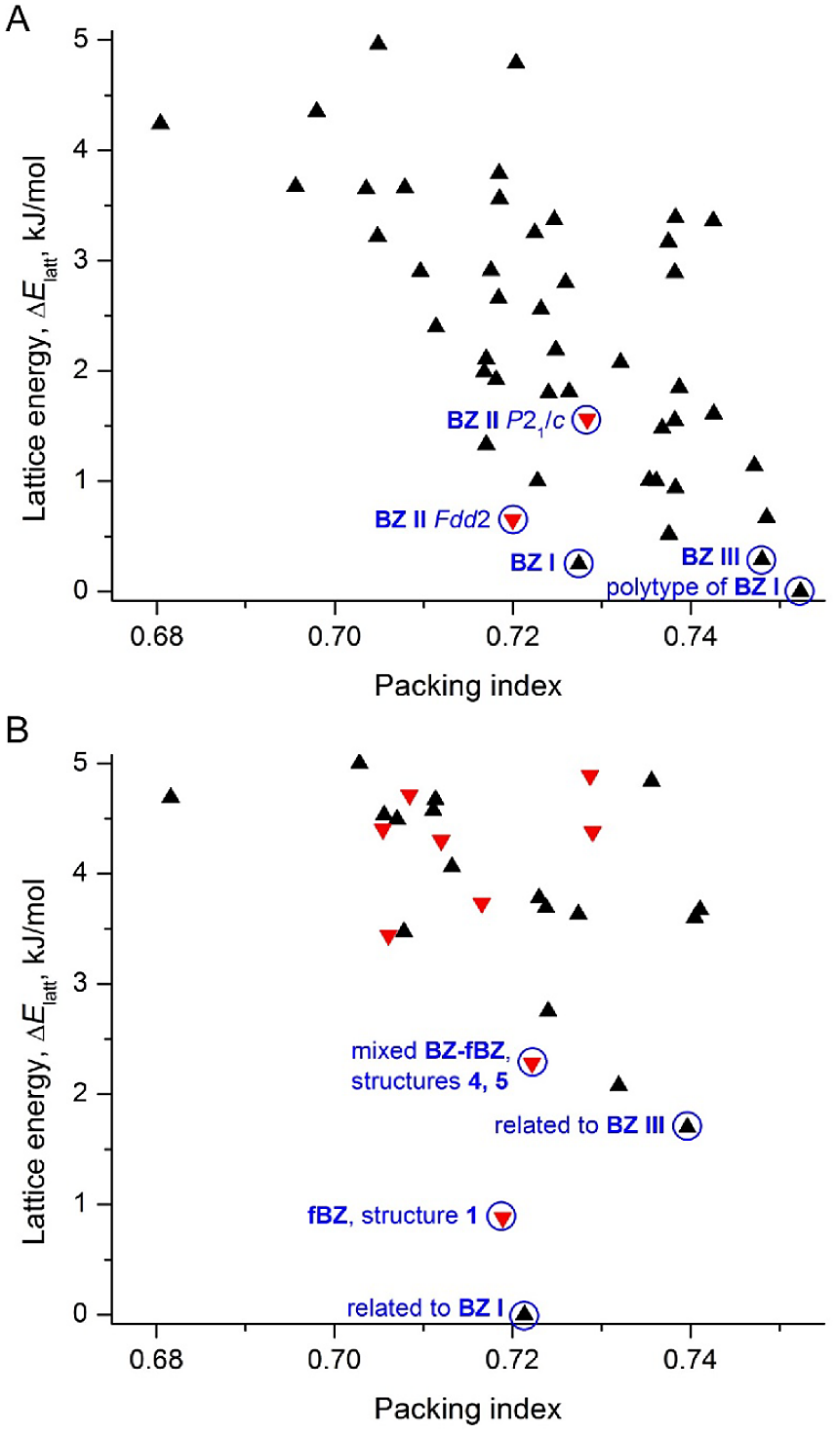
CSP landscape for benzamide (A) and 2-fluorobenzamide (B) with Δ*E*_latt_ ≤ 5.0 kJ mol^–1^. Red triangles correspond to structures adopting the double tape packing motif.

Benzamide and 2-fluorobenzamide crystal structures were generated by using CSP methods. They were optimized by DFT-*d* and ranked according to their lattice energy (Tables S1 and S2). For **BZ**, the experimental **I** and **III** crystal structures, as well as two previously predicted probable candidates for form **II** (*P*2_1_/*c* and *Fdd*2),^12,13,17^ were found within the lattice energy window Δ*E*_latt_ < 1.6 kJ mol^–1^ above the global minimum, which corresponded to a polytype of **I** (Figure 6A). For **fBZ**, the experimental structure (refcode BIGSUF and structure **2** in Table 2) was found with Δ*E*_latt_ = 0.88 kJ mol^–1^ above the global minimum, which corresponded to a structure like benzamide **I** but with a different orientation of the amide group (Figure 6B). The structures with the same packing as benzamide **III** (likely another observed **fBZ** polymorph, for which so far there is no crystal structure solution) and as a **fBZ-BZ** mixed crystal (structures **3** and **4** in Table 2) were found as well with Δ*E*_latt_ = 1.70 and 2.28 kJ mol^–1^, respectively (Figure 6B).

After removing duplicates and using a 5 kJ mol^–1^ energy cutoff, the CSP landscape of **BZ** and **fBZ** included 46 and 27 structures, respectively (Figure 6). Overall, **BZ** polymorphs heavily populated all energies while for **fBZ,** there is a clear gap up to Δ*E*_latt_ = 3.3 kJ mol^–1^, within which there were only six structures. Such an energy landscape suggests a smaller number of energetically accessible **fBZ** polymorphs compared to **BZ**.

For Δ*E*_latt_ < 3.3 kJ mol^–1^, only two structures with the double tape packing motif were predicted for both **BZ** and **fBZ**, and all of them correspond to experimental structures (Figure 6). For higher lattice energies (3.3 < Δ*E*_latt_ < 5 kJ mol^–1^), this packing motif was revealed only for **fBZ** (Figure 6). Previous CSP calculations,^12,17^ however, identified many such structures for **BZ** but most of them had Δ*E*_latt_ > 4.5 kJ mol^–1^. Thus, this analysis suggests that double-tape packing motifs can be found for both **BZ** and **fBZ** but would have relatively high lattice energies.

The packing similarity dendrograms^46^ (Figures S1 and S2), calculated without considering the F atom and the O and NH_2_ position of the amide, showed many structurally “identical” packing arrangements within the **BZ** and **fBZ** structures. Interestingly, the energy gaps between identical toluene fragments in the predicted structures were significantly smaller for **BZ** compared to **fBZ**. All **BZ** structures with identical toluene packings exhibited differences of <0.5 kJ mol^–1^, and six clusters were observed within the 5 kJ mol^–1^ range of the lowest energy structure. In contrast, the five clusters found within the same energy range for **fBZ** differed by 0.7, 0.8, 1.7–4.5, and 1.9 kJ mol^–1^ in *E*_latt_. Therefore, based on *E*_latt_ calculations, **BZ** could be inferred to be more susceptible to disorder than **fBZ**. Furthermore, cross-comparisons between the two sets of generated structures revealed numerous identical toluene arrangements within the **BZ** and **fBZ** structures, indicating the potential for mixed crystals.

Thus, as the fluorine content increases in both benzamide – 2-fluorobenzamide (**BZ-fBZ)** and thiobenzamide – 2-fluorothiobenzamide (**TBZ-fTBZ**) series, the complexity arising in disorder is resolved (Figure 2). The crystal structures of **fBZ** have simple organizations with *Z*’ = 2 and no disorder. The structures of **BZ-fBZ** solid solutions show more complex organization with *Z*’ = 4. Finally, **BZ** polymorphs **II** and **IV** show intergrowths of polytypes with different organizations of aryl rings with P vs V contacts and possibly in dimeric vs catemeric tapes. Likewise, the crystal structure of **fTBZ** and most **TBZ-fTBZ** solid solutions is simple with *Z*’ = 2 and similar to the structure of **fBZ**. The only difference is a minor disorder in orientation of phenyl rings, so that for **fTBZ**, one of two independent molecules exhibits *ca*. 15% of a conformer with the phenyl rings misoriented by 180°. The crystal structures close to **TBZ**, however, have a higher degree of complexity with *Z*’ = 4. They reveal disorder and strong twining with domains rotated 180° around the *z*-axis and the twin plane parallel to the short axis *b* ≈ 5.8 Å. The ability to grow large and more perfect crystals in both series also correlates with the degree of fluorination and disorder. Solid solutions close to **fBZ** and **fTBZ** can easily produce crystals suitable for single crystal X-ray diffraction analysis. For the intermediate compositions, it is still possible to grow single crystals, while for the compositions close to **BZ** and **TBZ** this does not seem possible.

Suppression of disorder at increasing fluorine concentration is curious because the desymmetrization of the phenyl ring by fluorine substitution is precisely the sort of perturbation that would be expected to lead to more disorder, as the least disorder with respect to aryl ring orientation, such as observed in **fTBZ**. The amount of fluorine necessary to achieve stabilization is fairly small, *x*_F_ < 0.3. It is worth noting that fluorination in other positions results in different packings. For example, the 3-fluorobenzamide packing (refcode BENAFM) is the same as in benzamide **III**,^47^ while the 3,5-difluorobenzamide packing (refcode APIHEO) is like benzamide **I**. Other fluorinated derivatives (4-fluorobenzamide (refcode BENAFP), 2,4-difluorobenzamide (refcode APIHAK), 2,3-difluorobenzamide (refcode APINUK), and 2,6-difluorobenzamide (refcode QEWGUW)) have unique crystal structures.

## Conclusions

We demonstrate that increasing the fluorine concentration in the continuous series of solid solutions of benzamide – 2-fluorobenzamide and thiobenzamide – 2-fluorothiobenzamide, which exhibit a double tape packing motif (Figure 1), suppresses disorder and enables growth of larger crystals without significantly altering the overall packing. Some explanation for the **BZ** and **fBZ** systems is provided by the computed lattice energy landscapes. Our results suggest that mono- and difluorination can be considered as a crystal engineering tool to perform fine-tuning of the crystal organization and control disorder.

## Supporting Information Available

List of CSP benzamide and 2-fluorobenzamide structures and packing similarity dendrograms (PDF)
