# Supplementary material for: Suppression of Disorder in Benzamide and Thiobenzamide Crystals by Fluorine Substitution
Source: Cryst Growth Des. 2024 Jun 10;24(12):5276–84. doi: 10.1021/acs.cgd.4c00517 (PMC11191397; doi:10.1021/acs.cgd.4c00517)
Supplement: Supplementary file 1 — cg4c00517_si_001.pdf [file cg4c00517_si_001.pdf]

Supporting Information

Suppression of disorder in benzamide and thiobenzamide crystals with fluorine substitution

Alexander G. Shtukenberg, Doris E. Braun, Melissa Tan, Noalle Fellah, Bart Kahr

1. Computational generation of fBZ and BZ crystal energy landscapes

Table S1. Computationally generated low-energy benzamide structures. The experimental structures are highlighted in green.

ID[a]	Space group	Cell parameters						ΔE_{latt}/ kJ mol^{-1}	PI
		a/Å	*b*/Å	*c*/Å	*α*/°	*β*/°	*γ*/°		
2	$P2_12_12_1$	5.073	5.508	42.547	90.00	90.00	90.00	0.00	0.752
1	$P2_1/c$	5.755	5.022	21.193	90.00	92.88	90.00	0.25	0.727
3	$P2_1/n$	5.062	5.372	21.962	90.00	94.47	90.00	0.29	0.748
6	$P2_1$	5.591	5.019	10.810	90.00	91.26	90.00	0.52	0.738
12	*Fdd*2	34.561	27.875	5.160	90.00	90.00	90.00	0.65	0.720
7	$P2_12_12_1$	5.058	5.499	43.061	90.00	90.00	90.00	0.67	0.749
5	$P2_1/c$	5.464	5.024	43.997	90.00	90.07	90.00	0.94	0.738
27	$Pna2_1$	11.600	5.180	20.262	90.00	90.00	90.00	1.00	0.736
4	$P2_1/c$	5.767	5.016	42.700	90.00	92.62	90.00	1.00	0.723
10	*P*-1	5.118	5.473	21.690	90.30	92.97	91.75	1.01	0.735
14	$P4_1$	5.115	5.115	45.731	90.00	90.00	90.00	1.14	0.747
30	$P2_1/c$	7.266	5.081	17.232	90.00	101.76	90.00	1.33	0.717
16	$P2_1/c$	5.476	5.082	43.536	90.00	90.36	90.00	1.48	0.737
34	$P2_1/c$	14.669	5.165	8.079	90.00	99.86	90.00	1.55	0.738
22	$P2_1/n$	5.232	18.242	12.885	90.00	93.64	90.00	1.56	0.728
13	*P*-1	5.100	5.225	22.751	92.97	94.82	91.09	1.61	0.743
17	$P2_1/n$	15.293	5.112	16.542	90.00	108.89	90.00	1.80	0.724
32	$P2_1$	7.378	5.084	8.378	90.00	102.40	90.00	1.81	0.726
28	$P2_1/c$	5.498	5.110	43.059	90.00	90.67	90.00	1.85	0.739
9	*P*-1	5.063	5.679	21.818	92.59	96.49	92.02	1.92	0.718
11	$P2_1/c$	5.702	5.056	43.297	90.00	92.27	90.00	1.99	0.717
35	$P2_1/c$	5.135	5.561	21.511	90.00	93.63	90.00	2.08	0.732
18	$P2_1/c$	5.664	5.064	43.431	90.00	92.11	90.00	2.11	0.717
20	$P2_1$	10.669	5.063	11.438	90.00	92.03	90.00	2.19	0.725
33	$P2_1/c$	5.691	5.098	21.571	90.00	92.64	90.00	2.40	0.711

25	$P2_1/c$	5.838	5.055	20.889	90.00	91.41	90.00	2.56	0.723
24	P-1	6.175	10.011	10.898	110.64	94.65	96.74	2.66	0.718
19	$Pna2_1$	9.656	25.162	5.072	90.00	90.00	90.00	2.80	0.726
41	$P2_12_12_1$	5.117	5.506	21.538	90.00	90.00	90.00	2.89	0.738
15	$P2_1/n$	12.466	5.026	19.959	90.00	91.12	90.00	2.90	0.710
31	$P2_1/c$	8.500	5.084	14.737	90.00	103.48	90.00	2.91	0.718
45	$P2_1/n$	5.234	19.587	6.214	90.00	107.46	90.00	3.17	0.737
38	$P2_1/c$	10.928	5.033	11.529	90.00	93.22	90.00	3.22	0.705
29	$P2_1/c$	14.695	5.118	16.726	90.00	101.62	90.00	3.25	0.722
50	$I2/c$	8.009	5.231	28.989	90.00	97.74	90.00	3.36	0.743
47	$P2_1/c$	14.428	5.171	8.296	90.00	96.97	90.00	3.37	0.725
44	$C2/c$	28.976	5.191	8.073	90.00	96.15	90.00	3.39	0.738
37	$P2_1$	5.638	5.106	10.842	90.00	91.87	90.00	3.56	0.718
42	$P2_1/c$	5.113	22.532	5.519	90.00	97.63	90.00	3.65	0.704
39	$P2_1$	7.536	5.097	8.449	90.00	104.27	90.00	3.66	0.708
43	$P2_12_12_1$	5.092	9.538	13.264	90.00	90.00	90.00	3.67	0.696
21	P21/*n*	15.275	5.106	16.111	90.00	97.59	90.00	3.79	0.718
40	$P2_1/c$	13.848	4.909	9.781	90.00	100.61	90.00	4.24	0.680
36	$P2_1/c$	9.318	7.092	9.717	90.00	96.00	90.00	4.35	0.698
46	$P2_1/c$	11.275	5.073	10.881	90.00	92.91	90.00	4.79	0.720
48	$P2_1/c$	10.647	6.020	9.928	90.00	94.53	90.00	4.96	0.705
49	P21/*n*	11.574	5.148	11.849	90.00	118.64	90.00	5.01	0.719

[a] ID according to *Cryst. Growth Des.* 2020, **20**, *4*, 2670–2682.

Table S2. Computationally generated low-energy 2-fluoro benzamide structures. The experimental structures are highlighted in green.

ID	Space group	Cell parameters						ΔE_{latt}/ kJ mol^{-1}	PI
		a/Å	b/Å	c/Å	α/°	β/°	γ/°		
1	$P2_1/c$	5.161	5.610	22.093	90.00	94.88	90.00	0.00	0.721
2	$P2_1/c$	5.177	20.489	12.345	90.00	96.40	90.00	0.88	0.719
3	$P2_1/n$	5.045	5.355	23.292	90.00	94.86	90.00	1.70	0.740
4	$P2_12_12_1$	5.055	5.550	22.697	90.00	90.00	90.00	2.08	0.732
5	$Pna2_1$	23.882	5.176	20.854	90.00	90.00	90.00	2.28	0.722
6	$P2_1/c$	5.587	5.006	45.816	90.00	91.10	90.00	2.75	0.724
7	$P2_12_12$	13.028	19.728	5.133	90.00	90.00	90.00	3.44	0.706
8	$P2_1/b$	5.673	5.035	23.044	90.00	95.03	90.00	3.47	0.708
9	P-1	5.186	5.227	23.395	93.30	96.26	91.30	3.60	0.740
10	$P2_1/c$	5.042	22.543	5.650	90.00	93.87	90.00	3.63	0.727
11	$P2_1/n$	10.314	5.414	22.940	90.00	101.38	90.00	3.67	0.741
12	$P2_12_12_1$	5.016	5.566	46.225	90.00	90.00	90.00	3.69	0.724
13	$P2_1/n$	5.093	20.281	12.694	90.00	97.01	90.00	3.73	0.717
14	$P2_1$	5.577	5.009	11.526	89.91	92.69	90.52	3.78	0.723
15	$P2_1/c$	5.598	5.000	46.544	90.00	92.27	90.00	4.06	0.713
16	$P2_12_12_1$	5.138	12.277	20.753	90.00	90.00	90.00	4.30	0.712
17	$P2_1/n$	5.146	20.641	12.212	90.00	98.76	90.00	4.38	0.729
18	$P2_1/n$	5.196	18.153	14.055	90.00	94.73	90.00	4.40	0.705
19	$P2_1$	5.692	5.022	11.564	90.00	95.15	90.00	4.49	0.707
20	$P2_1/c$	5.913	5.175	21.684	90.00	94.97	90.00	4.53	0.706
21	$Pna2_1$	5.010	46.936	5.564	90.00	90.00	90.00	4.57	0.711
22	$P2_1/c$	5.036	45.671	5.706	90.00	93.63	90.00	4.67	0.711
23	$P2_1/c$	4.998	14.140	9.563	90.00	92.99	90.00	4.69	0.682
24	$P2_1/c$	5.213	19.158	13.314	90.00	97.38	90.00	4.71	0.708
25	$P2_1/n$	5.223	5.163	46.963	90.00	93.08	90.00	4.84	0.736
26	$P2_1/c$	5.103	12.084	20.760	90.00	95.73	90.00	4.89	0.729
27	$P2_1/n$	5.087	11.466	11.481	90.00	97.25	90.00	5.00	0.703

2. Crystal packing similarity of the computationally generated structures

The packing similarity dendrograms were generated with the CCDC's 'packing_similarity_dendrogram' python script and standard settings available on GitHub (https://github.com/ccdc-opensource/csd-python-api-scripts/tree/main/scripts/packing_similarity_dendrogram) according to Childs et al. (*Crystal Growth & Design* **2009**, *9*, 1869-1888).

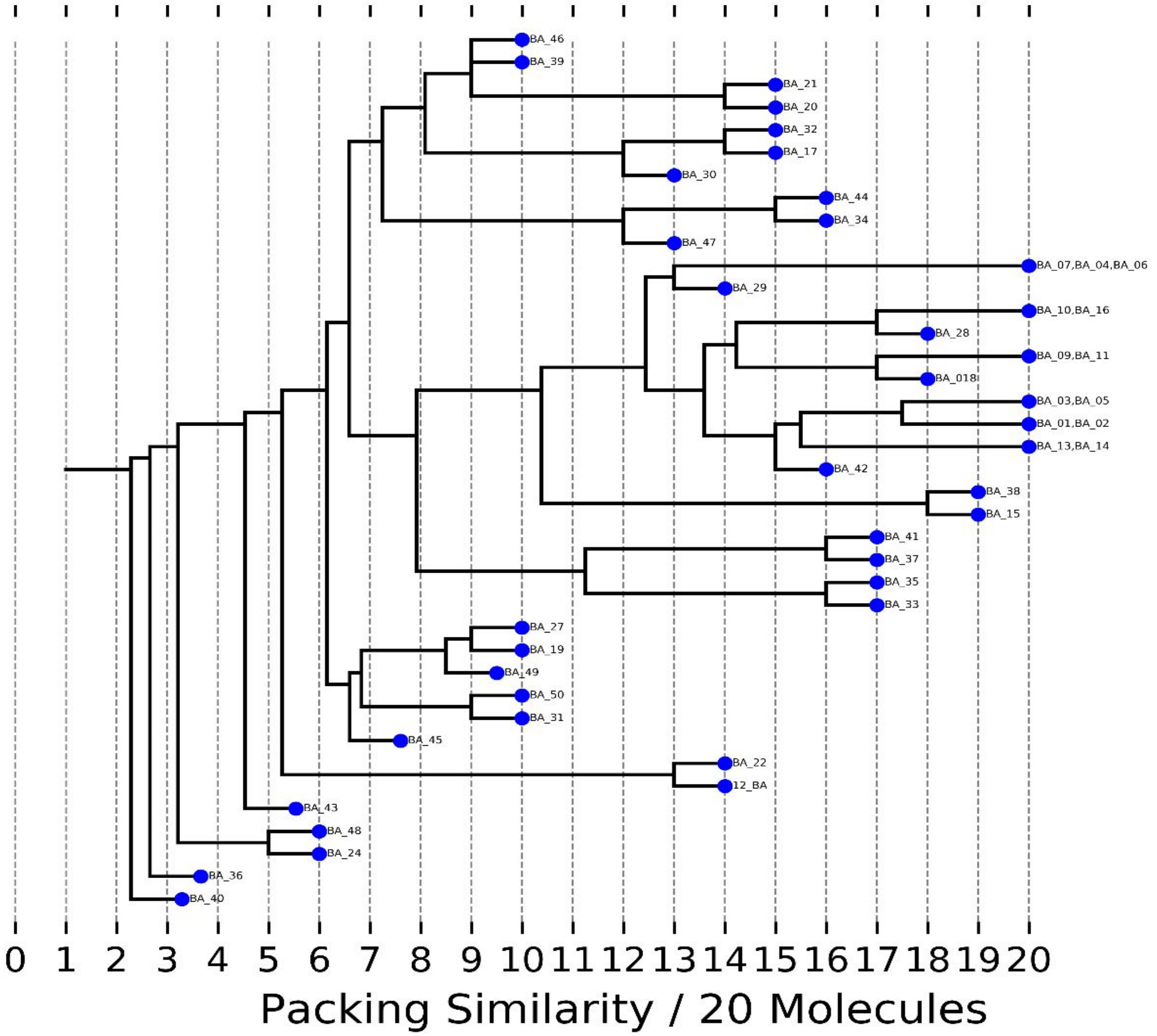

Figure S1. Packing similarity dendrogram of the computationally generated benzamide structures. The structure codes correspond to codes in Table S1.

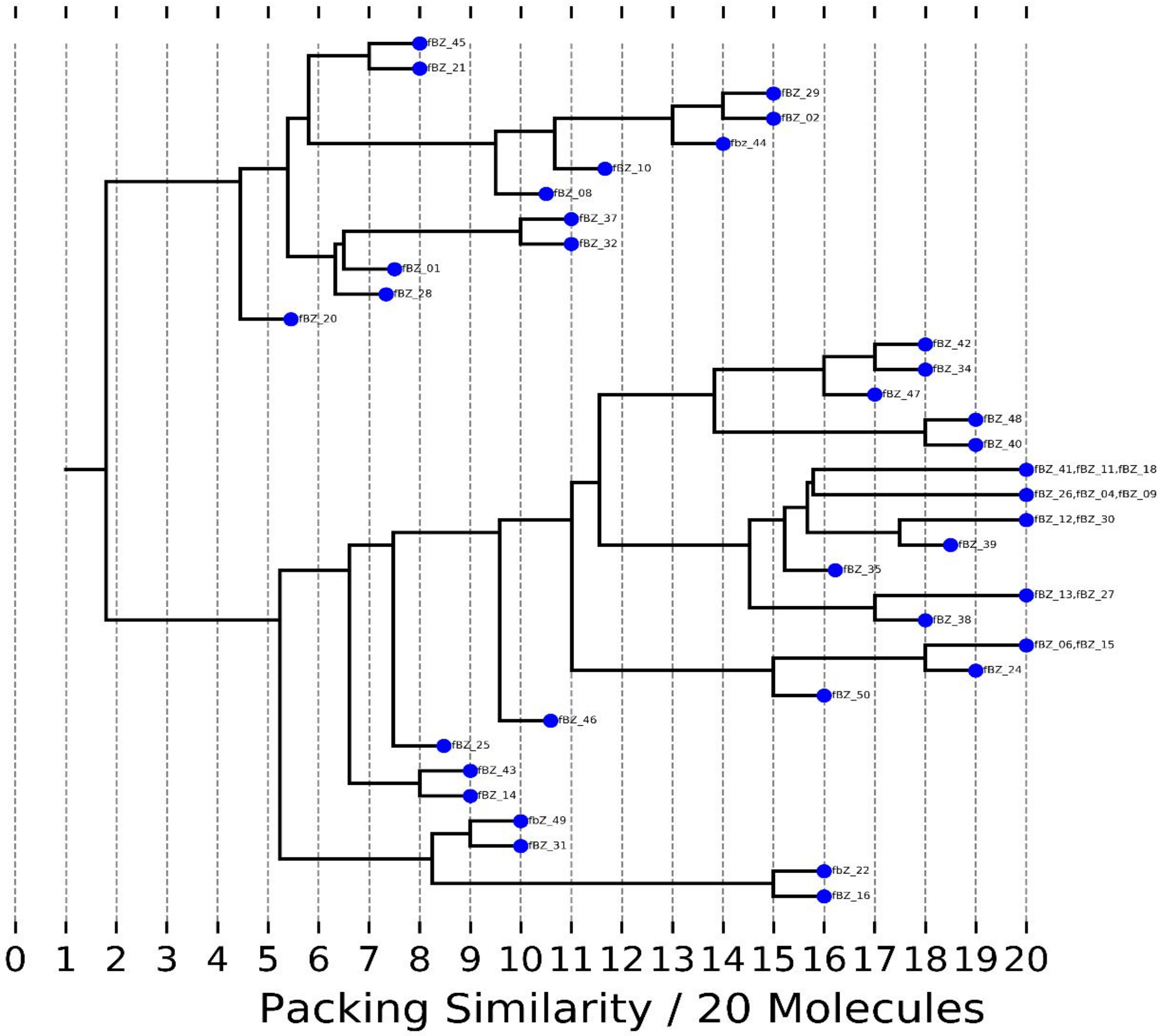

Figure S2. Packing similarity dendrogram of the computationally generated 2-fluoro benzamide structures. The structure codes correspond to codes in Table S2.
